# Evidence of endozoochory in upland geese *Chloephaga picta* and white‐bellied seedsnipes *Attagis malouinus* in sub‐Antarctic Chile

**DOI:** 10.1002/ece3.7725

**Published:** 2021-06-10

**Authors:** Xenabeth A. Lázaro, Roy Mackenzie, Jaime E. Jiménez

**Affiliations:** ^1^ Department of Wildlife Ecology and Conservation University of Florida Gainesville FL USA; ^2^ Sub‐Antarctic Biocultural Conservation Program Universidad de Magallanes Punta Arenas Chile; ^3^ Institute of Ecology and Biodiversity Santiago Chile; ^4^ Department of Biological Sciences and Advanced Environmental Research Institute University of North Texas Denton TX USA

**Keywords:** birds, bryophyte dispersal, endozoochory, mosses, sub‐Antarctic

## Abstract

Birds are known to act as potential vectors for the exogenous dispersal of bryophyte diaspores. Given the totipotency of vegetative tissue of many bryophytes, birds could also contribute to endozoochorous bryophyte dispersal. Research has shown that fecal samples of the upland goose (*Chloephaga picta*) and white‐bellied seedsnipe (*Attagis malouinus*) contain bryophyte fragments. Although few fragments from bird feces have been known to regenerate, the evidence for the viability of diaspores following passage through the bird intestinal tract remains ambiguous. We evaluated the role of endozoochory in these same herbivorous and sympatric bird species in sub‐Antarctic Chile. We hypothesized that fragments of bryophyte gametophytes retrieved from their feces are viable and capable of regenerating new plant tissue. Eleven feces disk samples containing undetermined moss fragments from *C. picta* (*N* = 6) and *A. malouinus* (*N* = 5) and six moss fragment samples from wild‐collected mosses (*Conostomum* *tetragonum*, *Syntrichia* *robusta*, and *Polytrichum* *strictum*) were grown ex situ in peat soil and in vitro using a agar Gamborg medium. After 91 days, 20% of fragments from *A. malouinus* feces, 50% of fragments from *C. picta* feces, and 67% of propagules from wild mosses produced new growth. The fact that moss diaspores remained viable and can regenerate under experimental conditions following the passage through the intestinal tracts of these robust fliers and altitudinal and latitudinal migrants suggests that sub‐Antarctic birds might play a role in bryophyte dispersal. This relationship may have important implications in the way bryophytes disperse and colonize habitats facing climate change.

## Graphical abstract

Birds are known to act as potential vectors for the exogenous dispersal of bryophyte diaspores. Given the totipotency of vegetative tissue of many bryophytes, birds could also contribute to endozoochorous bryophyte dispersal. We evaluated the role of endozoochory in two herbivorous and sympatric bird species in sub‐Antarctic Chile and hypothesized that fragments of bryophyte gametophytes retrieved from their feces are viable and capable of regenerating new plant tissue.

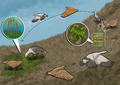

## 1 INTRODUCTION

Bryophytes are considered the direct descendants of the earliest forms of plants on Earth and are found anywhere from the tundra to the tropical rainforest. However, they are typically associated with temperate forests, peatlands, tundra, and alpine regions (Goffinet et al., 2012). With climate change as a growing concern for high‐elevation and high‐latitude habitats, and the large proportion of bryophyte endemism in high‐latitude regions (Rozzi et al., 2008), it is important to understand the dispersal mechanisms and potential for bryophytes to colonize new habitats. According to Urban (2015), if the Earth's temperature increases by 3℃, South America will be one of three regions where extinction risks of species will be highest (23%), followed by Australia and New Zealand (14%). Additionally, climate change is causing an upslope shift in montane plant and animal communities (Elsen & Tingley, 2015; Freeman et al., 2018) that is driven by niche conservatism, which is the retention of ancestral ecological characteristics, such as a habitat, by a species. Faced by climate change, species are more likely to respond by “following” their niches or ancestral climate regime rather than adapting their climatic tolerances (Wiens & Graham, 2005), which may represent a challenge for sessile organisms such as mosses.

Bryophytes, as most land plants, are sessile organisms depending on biotic or abiotic vectors to disperse propagules to other areas. Their movement is made possible via wind dispersal facilitated by sexual reproduction morphological features such as exposed spores, tall sporophytes, or the production of a large number of spores (Barbé et al., 2016; Muñoz, 2004); or via water dispersal, such as gemma or splash cups (Glime, 2017a, 2017b; Zanatta et al., 2018). On the other hand, some species of the Splachnaceae family, such as *Tayloria dubyi*, have even been known to have sticky spores and brightly colored sporophytes that emit a strong odor, mimicking decomposing organic matter, to attract flies as potential dispersal vectors (Jofre et al., 2011). Bryophyte dispersal and colonization are also facilitated by bryophyte cell totipotency, an asexual reproduction mechanism able to regrow the entire gametophyte from tissue fragments (Anderson, 1963; Cleavitt, 2002; Longton, 1997; Proctor et al., 2007; Zhang et al., 2003). However, because some birds and bryophytes share the same habitats, these birds may serve as animal vectors that allow these small plants to reach areas that they would not reach otherwise, or help reach them quicker and more frequently. The behavior of birds can aid in directed and long‐distance movement of bryophytes as they may act as dispersers in local sites through foraging and nesting (Amélio et al., 2017; Calvelo et al., 2006; Parnikoza et al., 2012, 2018), and transcontinentally through migratory movements (Chmielewski & Eppley, 2019). There has been evidence of migratory birds carrying bryophytes via ectozoochory, by external transportation (Lewis et al., 2014). Given this information, birds may be able to do the same via endozoochory, through internal ingestion, as has been shown with ferns and other herbs (Blanco et al., 2019; Hervías‐Parejo et al., 2019; Lovas‐Kiss et al., 2018; Silva et al., 2020).

The upland goose (*Chloephaga picta*, order Anseriformes, family Anatidae (Carboneras & Kirwan, 2020)) and the white‐bellied seedsnipe (*Attagis malouinus*, order Charadriiformes, family Thinocoridae (del Hoyo et al., 1996)) are two herbivorous bird species that inhabit sub‐Antarctic South America and could be potential vectors for bryophyte dispersal (Russo et al., 2020). *A. malouinus* is an altitudinal migratory shorebird that moves downwards from its upland habitats to lowland flats during the harsh winters (Fjeldså & Krabbe, 1990; del Hoyo et al., 1996; Jaramillo et al., 2003; “eBird: White‐bellied Seedsnipe *Attagis malouinus,*” n.d.) and has occasionally been known to leave Patagonia to the Falkland Islands (Hayman et al., 1986). *C. picta* is a migratory goose that is known to have larger movements through South America, migrating between breeding and wintering grounds. Pedrana et al. (2015) tracked the migratory route of a male *C. picta* and found that he migrated a minimum distance of 1,485 km from Buenos Aires (the wintering grounds at 34°S, 58°W) to Santa Cruz Province, Patagonia (the breeding grounds at 51°S, 69°W). This species also migrates altitudinally, as the same study found that the individual moved to lower than 100 masl on the wintering ground and between 1000–1500 masl on the breeding ground. Upland geese tagged on Navarino Island have been observed some 400 km north near Rio Gallegos in Argentina and some geese perform daily altitudinal migration from sea level up to over 700 masl there (J. Jiménez, 2020, pers. comm.).

Three moss species that are within the ranges of *C. picta* and *A. malouinus,* and that we hypothesize might be fed upon by them, are *Polytrichum strictum*, Brid., family Polytrichaceae, also known as “pigeon wheat”; *Syntrichia robusta* (Hook. & Grev.) R.H. Zander, family Pottiaceae; and *Conostomum tetragonum* (Hedw.) Lindb., family Bartramiaceae. *P. strictum* and *C. tetragonum* are bipolar species, meaning that they are present in high latitudes of both the Northern and Southern Hemispheres, whereas *S. robusta* is considered endemic to Patagonia (Pereira et al., 2006). According to the Tropicos database of the Missouri Botanical Garden (Tropicos.org, 2021, 2021, 2021; http://www.tropicos.org, visited on 4 April 2021), in the Southern Hemisphere, *P. strictum* can be found widely distributed from Bolivia to the Magallanes region of Chile and Tierra del Fuego, Argentina (Ireland et al., 2006). *C. tetragonum* extends from Costa Rica and Colombia (Ceballos & Rangel, 2008) to the Magallanes region. Finally, *S. robusta* was described between 35°S (Maule and Bío‐Bío, Chile) and 55°S (Magallanes, Chile, and Tierra del Fuego, Argentina). It can also be found within 40°S and 43°S in New Zealand and has been recorded in the Falkland and South Georgia Islands (Moraga, 2020; Pereira et al., 2006).

Previous research suggests that birds, such as mallard ducks (*Anas platyrhynchos*) and sub‐Antarctic geese and shorebirds, might be capable of dispersing bryophytes through endozoochory (Russo et al., 2020; Wilkinson et al., 2017). Additional evidence of bryophyte dispersal through endozoochory has been reported in spectacled flying foxes (*Pteropus conspicillatus*) (Parsons et al., 2007) and freshwater fish (Boedeltje et al., 2019). To our knowledge, the first observations of the consumption of bryophytes by *C*. *picta* and *A*. *malouinus* in sub‐Antarctic South America were made by Behling et al. (2016). More recently, Russo et al. (2020) observed that fecal samples collected on a drying snowmelt bed were comprised of about 50%–80% sporophyte fragments from the moss family Polytrichaceae, and those collected on flooded meadows were comprised of about 80%–100% bryophyte sporophytes. Of all fecal samples the authors uncovered, 91% of *C. picta* and 85% of *A. malouinus* samples contained bryophyte fragments, including fragments identified as *Polytrichum* sp., with at least one generating new growth.

Even though viable bryophyte gametophyte fragments have been recovered from avian and mammalian species that feed on these plants, previous research has attempted to cultivate or regenerate these fragments with little success (Parsons et al., 2007; Russo et al., 2020; Wilkinson et al., 2017). Given that the dispersal and establishment of plants involve sequential and interdependent steps to be successful under field conditions, it is inappropriate to assume that finding bryophyte fragments in bird feces is directly correlated with successful dispersal. Thus, we cannot conclude that these fragments successfully propagate after passing through the bird's digestive system. For these reasons, together with the search of viable fragments in bird feces, it was necessary to first test whether the bryophyte fragments found in bird feces were capable of regenerating when grown under laboratory conditions. Here, we report observations of the potential role of two herbivorous bird species, *C. picta* and *A. malouinus*, as endozoochorous bryophyte dispersers by testing the viability and regenerative capabilities of fragmented bryophyte gametophytes retrieved from their feces. We propose that both avian species have the potential to serve as dispersal vectors for bryophytes in the sub‐Antarctic through endozoochory. Our prediction is that after being ingested, defecated, and cultivated under the proper conditions, the bryophyte fragments will have the totipotence to regenerate a new individual. Endozoochory is likely to be a widespread phenomenon. Therefore, our findings could be applicable to regions beyond sub‐Antarctic Chile, like the Arctic, where birds also feed on mosses (Fox & Bergersen, 2005) and climate change is altering the vegetation communities and plant–herbivore relations (Bjorkman et al., 2018; Klein et al., 2008).

## 2 MATERIALS & METHODS

The research was conducted on Navarino Island (54°S, 67°W), Magellanic region, in sub‐Antarctic Chile, at the southern end of the Americas. The island has a rugged topography with marked, but still moderate seasons. At the lower ranges, the forest is covered by a mix of southern beech species (*Nothofagus betuloides* and *Nothofagus pumilio*) growing in krummholz formation at the tree line. Above the tree line, a rich community of small plants, including cushion plants, some graminoids, lichens, and mosses, thrive in a Magellanic tundra environment (Méndez et al., 2013). We sampled *C. picta* feces at sea level and *C. picta* and *A. malouinus* feces at ca. 700 masl, in open meadows some 100 m above the tree line. Fieldwork and laboratory work were conducted over the course of five weeks, and the growth of recovered fragments was monitored over an additional 13 weeks in a growth chamber in Navarino Island, from December 2018 to April 2019.

In the field, fresh *C. picta* and *A. malouinus* fecal samples were collected from six locations, focusing on upland and lowland sites. We sampled opportunistically two sites near sea level, Lake Zañartu (19F 587,064.87E 3,911,888.71S) and Robalo Bay (19F 585,969.41E 3,911,044.12S), and four sites above the tree line, near streams, and large ponds on the Cerro Bandera summit (19F 587,490.15E 3,908,430.45S, 19F 586,779.97E 3,907,237.18S, 19F 586,790.75E 3,907,270.36S). Fecal samples of both birds were identified in the field through our personal experience observing these birds in situ. Multiple fecal samples that visibly contained bryophyte fragments were collected from each site and later only one was selected from each site for processing (*n* = 6), three from each bird species. Of those six samples, there were two *C. picta* feces from lowland sites, one *C. picta* feces from an upland site, and three *A. malouinus* feces from upland sites. Additionally, three moss species (*P*. *strictum*, *S. robusta,* and *C*. *tetragonum*) were sampled near fecal sample sites to test for regeneration capabilities compared to fragments from fecal samples under the same growing conditions. Bryophytes collected in the field were identified by RM using the preliminary key to mosses of Isla Navarino (Buck & Goffinet, 2010).

In the laboratory, *C. picta* and *A. malouinus* fecal samples were stored in paper bags at room temperature before processing. We used a disinfected precision knife to remove the outer layer of the feces to eliminate the possibility of contamination by wind‐ or soilborne spores and fragments. Two thin disks were then sliced from each fecal sample (approximately 1 mm in thickness). Each disk was dissolved separately in clear dishes using filtered water collected from the Robalo River (0.22‐μm PVDF sterile syringe filters, Millipore, Cork, Ireland). The disk contents were observed under a Leica ICC50 HD compound microscope at 40x and 100x magnification. Bryophyte fragments were picked out from the dishes using a probe and forceps, placed into Eppendorf tubes corresponding to each disk sample and partially filled with filtered river water, following Russo et al.’s (2020) protocol. These samples were stored for a few days at 4℃ until inoculation. Additionally, the wild‐collected mosses sampled were also cut into fragments.

The six fecal samples (three from each bird species) resulted in 11 disk samples analyzed, and 138 bryophyte fragments recovered and inoculated (106 belonging to *C. picta* and 32 to *A. malouinus* samples). The treatments tested were either a culture container, consisting of two plastic cups sealed together at the openings with parafilm to obtain a greenhouse effect, with commercial sphagnum peat soil (Kekkilä Professional Substrate, Vantaa, Finland) or a microplate for in vitro growth. The in vitro treatment was made using an agar Gamborg mixture basal salt (B5 salts) medium (G768, Phytotechnology Laboratories) with a pH of 5.8, a specific medium for moss growth (González et al., 2006). Once all fecal samples were processed, we randomly assigned the fragments from each fecal disk sample and from each moss species sample to either treatment to be propagated, with the exception of one *A. malouinus* sample, which only had one disk that contained one bryophyte fragment, and was therefore placed only in the agar Gamborg treatment. For the peat soil treatment, the fragments were placed on the soil, thoroughly misted with filtered water, and then covered and sealed for the remainder of the experiment with occasional misting throughout the observation period to keep the culture containers humid. High inner humidity and condensation were observed in the sealed containers throughout the experiment. For the agar Gamborg treatment, the samples were placed in a 28‐well agar plate and then covered and sealed with parafilm. A total of 18 culture mediums were placed into a growth chamber, with the temperature fluctuating between a minimum of 5 ºC and maximum of 25 ºC throughout the entirety of the experiment. Bryophyte cultures were grown on red and blue LED lights in a 4:1 ratio, at a day:night cycle of 16:8 hr. The wild moss sample fragments inoculated in the peat soil containers were placed in the growth chamber on 8 January 2019, followed by the fecal sample fragments inoculated in the peat soil containers on 9 January 2019, and fecal sample and wild moss sample fragments inoculated in the agar Gamborg on 11 January 2019. All the samples were left in the growth chamber for a total of 91 days.

## 3 RESULTS AND DISCUSSION

**FIGURE 1:**
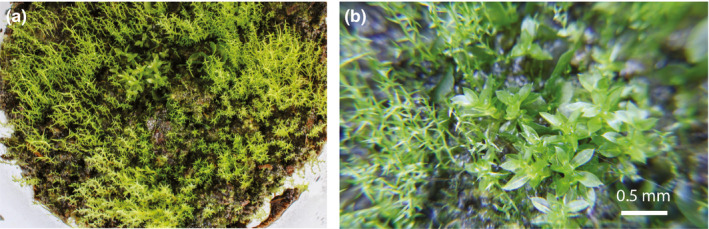
Moss regeneration from bryophyte fragments. Detailed image of green shoots and a moss bed that regenerated in the peat soil treatment from the bryophyte fragments extracted from an upland goose (*Chloephaga picta*) fecal sample

A total of 8 culture mediums of 18 (44%) produced growth in the treatments tested. One of five culture mediums with *A. malouinus* fragment samples (20%), three of six with *C. picta* fragment samples (50%), and four of six with wild moss fragment samples (67%) produced new growth (Table 1) based on the occurrence of light‐colored green shoots and moss beds (Figure 1). Five of the nine (56%) fecal fragment inoculations treated to the peat soil treatment (three from *C. picta*, one from *A. malouinus*, and one from *Polytrichum* sp.) and all three (100%) inoculations of fragmented wild mosses in the solid agar Gamborg medium showed vegetative growth. On the contrary, no growth was observed in the agar Gamborg for any of the six inoculations tested from the feces of both bird species.

**TABLE 1:** Summary of bryophyte regeneration results for each bird fecal and wild moss sample in each treatment

Sample	Peat Soil	Agar Gamborg
*Chloephaga* feces 1	+	‐
*Chloephaga* feces 2	+	‐
*Chloephaga* feces 3	+	‐
*Chloephaga* TOTAL (%)	100	0
*Attagis* feces 1	‐	‐
*Attagis* feces 2	NA	‐
*Attagis* feces 3	+	‐
*Attagis* TOTAL (%)	50	0
*Syntrichia*	‐	+
*Polytrichum*	+	+
*Conostomum*	‐	+
MOSS TOTAL (%)	33	100

Note

Growth results for fragments from feces and mosses (*N* = 17). Regeneration presence and absence of fragments recovered from upland goose (*Chloephaga picta*) and white‐bellied seedsnipe (*Attagis malouinus*) feces and fragments of wild‐collected mosses (*Syntrichia robusta*, *Polytrichum strictum*, and *Conostomum tetragonum*) grown under controlled light regimes and two types of substrates in a growth chamber.

Symbols and acronyms for samples indicate *Chloephaga*: *C*. *picta* (upland goose); *Attagis: A*. *malouinus* (white‐bellied seedsnipe); *Syntrichia: S*. *robusta* moss sample; *Polytrichum: P*. *strictum* moss sample; *Conostomum: C*. *tetragonum* moss sample; +: growth; ‐: no growth; NA: no sample in that combination.

Previous research has attempted to cultivate fragments found in feces in vitro with some success (Russo et al., 2020; Wilkinson et al., 2017). In this study, we partially followed Russo et al.’s (2020) fragment‐processing methods. However, we decided to set two growing conditions for the fragments recovered from the feces to increase the probability of bryophyte growth. These consisted of an ex situ growth condition using commercial peat soil and an in vitro condition using agar Gamborg medium for both fragments from fecal and wild moss samples taken from the field. We predicted that the fragments from both wild moss and fecal samples would regenerate. Previous research described growth from bryophyte spores after 60 days (Proctor, 1961) and from bryophyte fragments after 11 days (Wilkinson et al., 2017). However, we did not observe signs of regeneration from our cultures until 42 days of growth.

Our reasoning for the chosen treatments was to test which, if any, would allow bryophyte fragment recovery and growth under laboratory conditions. Although the sample size was small, we observed clear evidence of bryophyte regeneration from feces of both bird species in the peat soil treatment (Table 1). These results reveal that a small fraction of moss diaspores remains viable following the passage through the intestinal tract of both birds and is capable of regeneration in suitable conditions. Although small, our cases are significant. Given the volume of the feces and the number of feces defecated daily by each bird, if our results were multiplied by the number of birds per area and time, our results would translate into a large absolute number. Additionally, the growth evidence observed in the three wild mosses in the agar Gamborg confirmed that the bryophytes sampled are totipotent, which is a necessary condition for effective endozoochory mechanisms.

It is plausible that the in vitro (agar Gamborg) conditions could have yielded false‐negative results from the fecal sample fragments tested of both bird species (see Table 1), as moss diaspores may take longer to germinate in sterile conditions due to the necessary acclimation to the agar Gamborg substrate and near‐neutral pH conditions in the medium (Sabovljevic et al., 2014). However, there was evidence of bryophyte regeneration in fragments recovered from feces in the peat soil substrate. On the other hand, wild moss gametophyte growth was observed in samples inoculated both in agar Gamborg medium and peat soil. Therefore, these results support the hypothesis of bird endozoochory of these bryophytes in the sub‐Antarctic environment.

## 4 CONCLUSIONS

**FIGURE 2:**
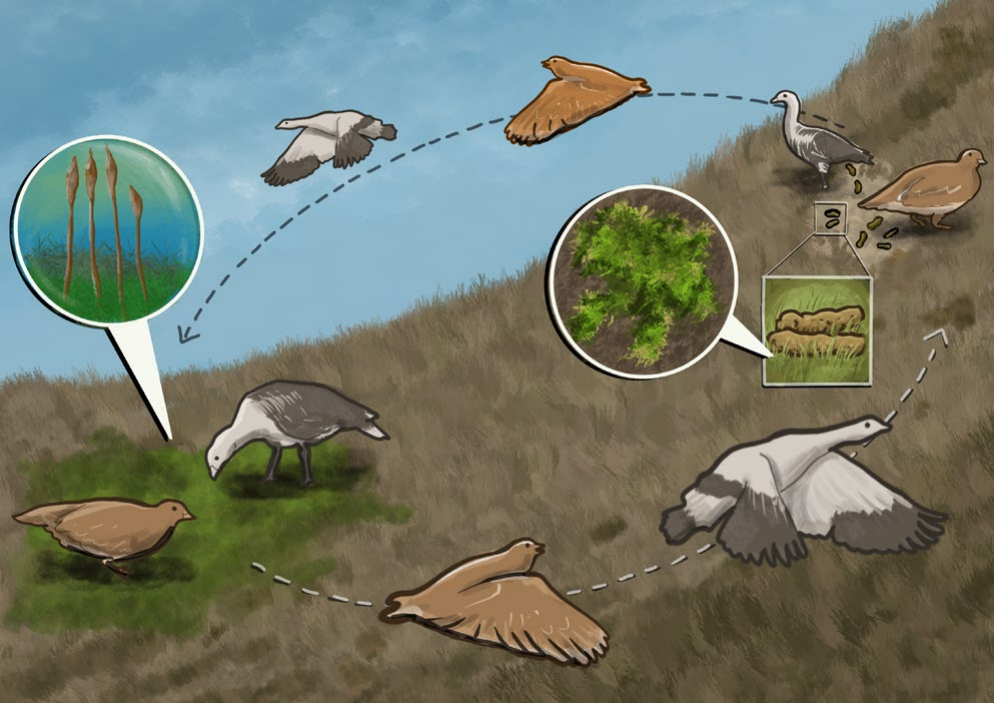
Schematic diagram of native bryophyte endozoochory by sub‐Antarctic birds. Schematic illustration of the possible endozoochory mechanism of bryophyte dispersal by the upland goose (*Chloephaga picta*) and the white‐bellied seedsnipe (*Attagis malouinus*)

We confirmed the hypothesis that fragmented bryophyte gametophytes retrieved from the feces of herbivorous birds could regenerate in laboratory conditions. Consequently, our research shows that it is plausible for fragments to be dispersed through endozoochory by these herbivorous birds in the sub‐Antarctic, possibly further aiding bryophytes in this region to disperse beyond wind or rain, and increasing their capability to reach specific habitat types (Figure 2). As both bird species are robust fliers, altitudinal migrants for short distances and latitudinal migrants for long distances, their role as dispersers might not only occur at a local scale within high‐latitude regions, but also at a broader scale to similar habitats across latitudes where these bryophyte species are still found. This is especially important as with warming conditions, organisms such as mosses would have to move to higher altitudes or latitudes to maintain viable populations. By serving as dispersal vectors, birds would be able to aid in this process in short time scales in the vertical and horizontal axes of the landscape. This process is especially critical in maintaining the viability of tundra bryophytes in Navarino Island, which contains an ecosystem of high ecological importance (Goffinet et al., 2012) and holds many native species (Méndez et al., 2013), and just as critical globally where bryophytes are also being affected by changes to their ecosystems.

Sub‐Antarctic birds may play a passive, but likely critical role in the dispersal of bryophytes, as birds are more likely to defecate in like habitat conditions where they graze (J. Jiménez, 2019, pers. comm.), potentially enabling bryophytes to effectively propagate within a generally suitable habitat and in locations where they are unlikely to be established by wind or rain, but are accessible to birds. However, further research needs to be done to test bryophyte dispersal through endozoochory and the role of birds in this process. To progress along this line of research, we suggest a larger sample size and replication, more substrate treatments, as bryophyte fragments might respond variably to distinct types of soil, and conducting DNA sequencing of the bryophyte fragments found in the fecal samples to identify the bryophyte species before culturing them. Another suggestion would be to analyze any genetic variations within bryophyte populations in relation to their altitudinal distributions and compare them to areas inside and outside of the migratory routes of *C. picta* and *A. malouinus*. This would further demonstrate whether the birds are dispersing the bryophytes and connecting bryophyte populations across different geographic regions. Finally, there is the need to reproduce these results under field conditions, so we can understand the relevance of this type of zoochory in bryophyte dispersal at these high‐latitude landscapes.

## CONFLICT OF INTEREST

The authors declare no competing interests.

## AUTHOR CONTRIBUTION

**Xenabeth Ashley Lazaro:** Conceptualization (equal); Investigation (lead); Methodology (equal); Writing‐original draft (lead); Writing‐review & editing (equal). **Roy Mackenzie:** Conceptualization (equal); Data curation (equal); Methodology (equal); Supervision (supporting); Writing‐review & editing (equal). **Jaime Jimènez:** Conceptualization (equal); Funding acquisition (lead); Methodology (equal); Supervision (supporting); Writing‐review & editing (equal).

## Data Availability

The raw data used in the present study are publicly available in the Dryad repository at https://doi.org/10.5061/dryad.ghx3ffbmm
